# Association between *ST8SIA2* and the Risk of Schizophrenia and Bipolar I Disorder across Diagnostic Boundaries

**DOI:** 10.1371/journal.pone.0139413

**Published:** 2015-09-29

**Authors:** So Yung Yang, Ik Soo Huh, Ji Hyun Baek, Eun-Young Cho, Mi Ji Choi, Seunghyong Ryu, Ji Sun Kim, Taesung Park, Kyooseob Ha, Kyung Sue Hong

**Affiliations:** 1 Department of Psychiatry, Sungkyunkwan University School of Medicine, Samsung Medical Center, Seoul, Korea; 2 Department of Statistics, Seoul National University, Seoul, Korea; 3 Center for Clinical Research, Samsung Biomedical Research Institute, Seoul, Korea; 4 Seoul National Hospital, Seoul, Korea; 5 Department of Neuropsychiatry, Seoul National University College of Medicine, Seoul National University Bundang Hospital, Kyunggi-Do, Korea; CNRS UMR7275, FRANCE

## Abstract

**Background:**

Findings from family studies and recent genome-wide association studies have indicated overlap in the risk genes between schizophrenia and bipolar disorder (BD). After finding a linkage between the *ST8SIA2* (ST8 alpha-N-acetyl-neuraminide alpha-2, 8-sicalyltransferase 2 gene) locus (15q26) and mixed families with schizophrenia and BD, several studies have reported a significant association between this gene and schizophrenia or BD. We investigated the genetic association between *ST8SIA2* and both schizophrenia and BD in the Korean population.

**Methods:**

A total of 582 patients with schizophrenia, 339 patients with BD, and 502 healthy controls were included. Thirty-one tag single nucleotide polymorphisms (SNPs) across the *ST8SIA2* region and three other SNPs showing significant associations in previous studies were genotyped. The associations were evaluated by logistic regression analysis using additive, dominant, and recessive genetic models.

**Results:**

Fourteen of 34 SNPs showed a nominally significant association (*p* < 0.05) with at least one diagnostic group. These association trends were strongest for the schizophrenia and combined schizophrenia and bipolar I disorder (BD-I) groups. The strongest association was observed in rs11637898 for schizophrenia (*p* = 0.0033) and BD-I (*p* = 0.0050) under the dominant model. The association between rs11637898 and the combined schizophrenia and BD-I group (*p* = 0.0006, under the dominant model) remained significant after correcting for multiple testing.

**Discussion:**

We identified a possible role of *ST8SIA2* in the common susceptibility of schizophrenia and BD-I. However, no association trend was observed for bipolar II disorder. Further efforts are needed to identify a specific phenotype associated with this gene crossing the current diagnostic categories.

## Introduction

Diagnoses of major psychiatric illnesses are based on phenomenological characteristics, resulting in an overlap in symptoms and treatment between diagnostic categories, as well as an overlap in genetic and biological risk factors [1]. Since the Kraepelinian dichotomy was proposed, schizophrenia and bipolar disorder (BD) have been classified as two distinct clinical entities based on their symptom patterns and course of illness [2]. However, common clinical features, such as psychotic symptoms, emotional dysregulation, and cognitive impairment have been described across the two disorders [3–6]. Findings from genetic studies also suggest shared familial risk and genetic susceptibilities between schizophrenia and BD [1, 7, 8].

Chromosome locus 15q26 has been identified as a candidate region for both schizophrenia and BD in a whole-genome linkage study of eastern Quebec families with combined schizophrenia and BD [9]. This locus has also been reported as a susceptibility locus for BD in a genome-wide linkage analysis in Australian multi-generational families with BD and a broad spectrum of clinical diagnoses, including major depressive disorder and schizoaffective disorder-manic type [10]. *ST8SIA2* (ST8 alpha-N-acetyl-neuraminide alpha-2, 8-sicalyltransferase 2 gene) is one of the candidate genes for psychiatric illnesses mapping to this region. In a recent genome-wide association study (GWAS) of Chinese patients with BD, the most strongly associated single nucleotide polymorphisms (SNPs) were close to *ST8SIA2* with the lowest *p*-value of 4.87 × 10^−7^ [11].

Targeted gene studies for *ST8SIA2* have been performed in patients with schizophrenia. Three SNPs (rs3759916, rs3759915, and rs3759914) located in the promoter region show a significant association with schizophrenia in the Japanese (rs3759916 and rs3759914) [12] and Chinese (rs3759915) [13] populations. rs3759916 also shows a sex-specific association with schizophrenia in female Spanish patients with schizophrenia [14]. A BD association study was performed in the Australian population [15] as a fine mapping study of their previous linkage finding [10] in families with BD. In that study, a number of SNPs (rs4586379, rs2035645, rs4777974, rs11637898, rs11074070, rs3858917, rs3784735, and rs2168351) showed a nominally significant association with bipolar spectrum disorders, and a specific risk haplotype was identified. The authors also observed an over-representation of this risk haplotype in an Australian schizophrenia case-control cohort. The same group identified two variants (rs11074064 and rs722645) with putative functional effects that were nominally associated with BD more recently [16].

*ST8SIA2* encodes polysialyl transferase, which is involved in the biosynthesis of polysialic acid (polySia) that spatiotemporally modifies neural cell adhesion molecule (NCAM) [17]. The polySia-NCAM complex is predominantly found in the embryonic brain, and its expression in the adult brain is highly restricted to areas where neural plasticity is ongoing, such as the hippocampus, subventricular zone, thalamus, prefrontal cortex, and amygdala [18]. The polySia-NCAM complex is thought to regulate neuronal processes, such as neuronal migration [19] and synaptogenesis [20]; thus, anomalous expression of polySia impairs spatial learning and memory [21–23]. Since growing evidence suggests that a neurodevelopmental deficit is important in the pathophysiological mechanism of schizophrenia and BD [24, 25], *ST8SIA2* could be a plausible functional candidate gene.

Our group performed a genome-wide linkage scan of quantitative traits targeting symptom dimensions in multiplex schizophrenia families [26]. The 15q26 locus harboring *ST8SIA2* showed a linkage signal that attained the genome-wide empirical threshold for suggestive linkage with the “non-paranoid delusion factor”. Interestingly, lifetime symptoms for this factor include grandiose, religious, and erotic delusions that are frequently observed in patients with schizophrenia or BD [26]. Based on these findings and previous studies of other populations indicating that *ST8SIA2* is a candidate gene for both schizophrenia and BD, the current study investigated the genetic association between *ST8SIA2* and schizophrenia and BD in the Korean population. We analyzed tag SNPs covering the whole gene locus and applied genotype-based analyses.

## Materials and Methods

### Study Subjects

Patients who met the DSM-IV [27] diagnostic criteria for schizophrenia (*n* = 582) and BD (*n* = 339), including bipolar I disorder (BD-I) (*n* = 180) and bipolar II disorder (BD-II) (*n* = 159) were recruited from the inpatient unit and outpatient clinic of Samsung Medical Center and Seoul National University Bundang Hospital.

The healthy subjects (*n* = 502) consisted of volunteers from the community who were free of any history of clinically significant psychiatric symptoms. Written informed consent was obtained from all subjects after a complete explanation of the study. This study was approved by the institutional review boards of Samsung Medical Center and Seoul National University Bundang Hospital.

### SNP Selection

We used the Korean Hapmap database (http://www.khapmap.org) to select *ST8SIA2* tag SNPs. Thirty-one tag SNPs were chosen by the program Tagger within Haploview v4.0 (http://www.broad.mit.edu/mpg/haploview) using pairwise tagging of SNPs with an r^2^ > 0.8 and minor allele frequency (MAF) > 0.05 [28, 29]. An additional three SNPs (rs3759916, rs3759915, and rs3759914), which previously showed a significant association with schizophrenia [12, 13] in other Asian populations, were included in the analysis. The selected SNPs and their genomic or intragenic location, allele types, and minor allele frequencies are summarized in Table 1. All SNPs were intronic, except rs3759916, rs3759915, and rs3759914 on the 5′-untranslated region (UTR) and rs2290492 and rs17600420 on the 3′-UTR. These SNPs spanned the entire *ST8SIA2* gene with an average inter-SNP distance of 2.2 kb (range, 56 bp–12.7 kb).

**Table 1:** Characteristics of the 34 *ST8SIA2* tag SNPs.

SNP	Genomic location	Intragenic location	Alleles	MAF ^a^	SNP	Genomic location	Intragenic location	Alleles	MAF ^a^
rs3759916	Chr15:90737173	5’-UTR	A>G	0.36	rs3784732	Chr15:90787919	intron 4	T>C	0.03
rs3759915	Chr15:90737392	5’-UTR	G>C	0.48	rs3784731	Chr15:90788082	intron 4	A>T	0.29
rs3759914	Chr15:90737448	5’-UTR	A>G	0.35	rs1455777	Chr15:90789744	intron 5	T>C	0.29
rs4777969	Chr15:90741567	intron 1	G>A	0.37	rs7166344	Chr15:90791345	intron 5	G>A	0.30
rs8025225	Chr15:90741903	intron 1	T>C	0.39	rs1487982	Chr15:90795915	intron 5	T>C	0.14
rs2124359	Chr15:90743092	intron 1	G>C	0.49	rs1352323	Chr15:90796644	intron 5	C>T	0.34
rs1487984	Chr15:90746120	intron 1	C>A	0.11	rs3784729	Chr15:90797010	intron 5	C>T	0.30
rs11074067	Chr15:90747301	intron 1	G>C	0.43	rs10775256	Chr15:90798158	intron 5	A>G	0.36
rs881770	Chr15:90750812	intron 1	G>A	0.49	rs897463	Chr15:90801444	intron 5	T>A	0.09
rs4777973	Chr15:90751886	intron 1	A>G	0.34	rs11852344	Chr15:90802524	intron 5	A>G	0.49
rs4777974	Chr15:90753293	intron 1	A>G	0.11	rs4777715	Chr15:90803084	intron 5	A>G	0.39
rs7176813	Chr15:90753636	intron 1	A>G	0.40	rs4777988	Chr15:90803447	intron 5	A>G	0.43
rs11637898	Chr15:90753853	intron 1	A>G	0.25	rs3784723	Chr15:90806762	intron 5	T>C	0.36
rs4777980	Chr15:90766580	intron 1	A>G	0.28	rs4777989	Chr15:90807743	intron 5	A>G	0.46
rs3784737	Chr15:90768555	intron 1	A>G	0.29	rs7168443	Chr15:90808052	intron 5	C>T	0.09
rs8037133	Chr15:90775460	intron 2	A>G	0.29	rs2290492	Chr15:90808977	3’-UTR	C>T	0.11
rs2168351	Chr15:90784725	intron 4	C>T	0.33	rs17600420	Chr15:90812138	3’-UTR	A>G	0.40

SNP, single nucleotide polymorphism; MAF, minor allele frequency

^a^ Minor allele frequency based on the control group data

### DNA Extraction and Genotyping

Whole blood was collected from each individual into EDTA tubes, and genomic DNA was isolated from peripheral blood leukocytes using the Wizard Genomic DNA Purification kit according to the manufacturer’s instructions (Promega, Madison, WI, USA). MassARRAY Assay Design, version 3.0 software (Sequenom Inc, San Diego, CA, USA) generated three multiplex reactions: 12 SNPs (plex 1), 12 SNPs (plex 2), and 10 SNPs (plex 3). Multiplex SNP genotyping was performed by primer extension and matrix-assisted laser desorption/ionization time-of-flight mass spectrometry using the iPLEX Gold technology from Sequenom. SNP assays were designed using Sequenom MassARRAY Assay Design ver. 3.0 software (primer information is available upon request). The polymerase chain reaction was performed according to the standard iPLEX methodology. Spectra were analyzed by MassARRAY Typer ver. 3.4 software (Sequenom). Quality control was performed to exclude individual SNPs or samples with genotype call rates < 95% and SNP assays with poor-quality spectra/cluster plots.

To confirm the reliability of iPLEX SNP genotyping method, five selected SNPs (rs4777973, rs3784731, rs11637898, rs1455777, and rs897463) for 48 random samples were regenotyped by sequencing reaction using ABI PRISM BigDye Terminator v 3.1 Cycle Sequencing Kits (Applied Biosystems, CA, USA) The concordance rate of genotype data between sequencing and iPLEX SNP genotyping was 99.2%

### Statistical Analyses

We used the Kruskal–Wallis test or the chi-square test to reveal differences in demographic variables among the patient and control groups. The Hardy–Weinberg equilibrium was checked with Fisher’s exact test for the genetics analysis, and no significant deviation was observed in any of the SNPs (S1 Table). Genotype-wise association was evaluated by logistic regression analysis with age and sex as covariates. Additive, dominant, and recessive genetic models were considered based on the minor allele of each SNP. The inheritance model with the least Akaike Information Criterion [30] was accepted as the best fitting model. We controlled the experiment-wise type I error using the Bonferroni correction. Thirty-four SNPs were analyzed; thus, a *p*-value = 0.0015 (0.05/34) was the adjusted level of significance. All statistical analyses were done with snpStats ver. 1.18.0 in R ver. 3.0.2 (http://www.bioconductor.org) [31].

## Results

The demographic and clinical characteristics of the subjects are presented in Table 2. More males were present in the schizophrenia group than in the BD-I and control groups, and the BD-II group had more females than in the other groups. The patients with BD-II were older than those in the other groups, and subjects in the control group were younger than the patients.

**Table 2:** Demographic characteristics of the subjects.

Demographic characteristics	Control (N = 502)	Schizophrenia (N = 582)	BD-I (N = 180)	BD-II (N = 159)	Statistics ^a^	
Age, mean(SD)	31.6 (7.9)	33.9 (9.5)	33.9 (10.1)	37.4 (11.4)	F = 16.6	P<0.001
Sex (Male, %)	43.8	53.6	38.3	25.2	χ2 = 46.2	P<0.001

BD-I, bipolar I disorder; BD-II, bipolar II disorder

^a^ After post-hoc analysis, BD-II group was older than the other groups and the control group was younger than the other groups. More males were in the schizophrenia group than in the BD-1 and control groups, and the BD-II group had more females than the other groups.

All SNPs passed quality control with MAF > 0.01, and the missing data rate for each SNP was < 2%. Therefore, all of the genotyped SNPs (*n* = 34) were included in the statistical analysis.

**Fig 1:**
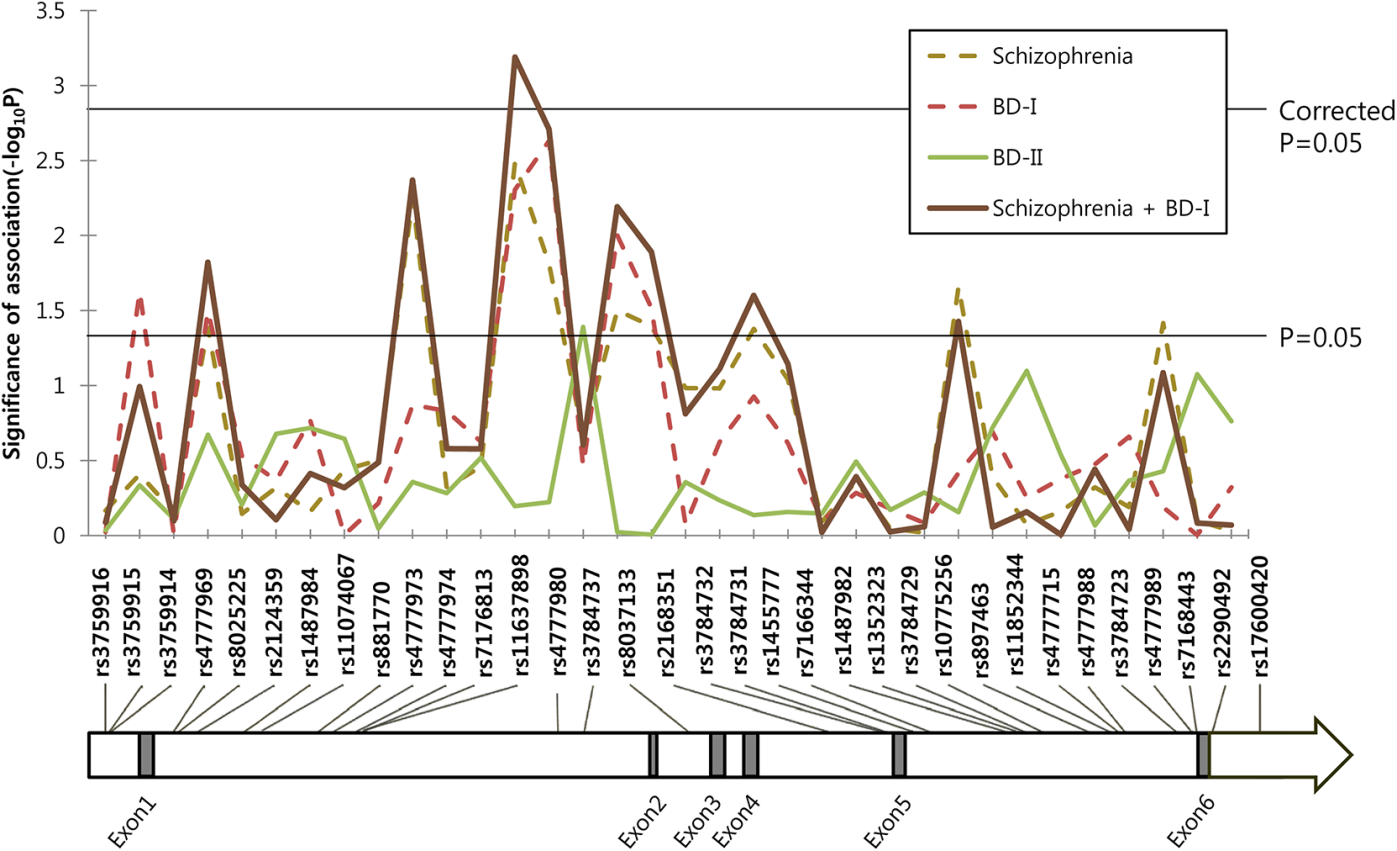
Association pattern between *ST8SIA2* tag single nucleotide polymorphisms (SNPs) and schizophrenia and bipolar disorder under the dominant model. –Log of *p*-values are represented on the y-axis with relative location of the SNPs in the gene on the x-axis. Two horizontal lines indicate nominal and corrected *p*-values of 0.05.

The overall SNP-disease association plot is shown in Fig 1, and the level of significance is shown as –log *p*-value. Schizophrenia and BD-I showed similar association trends throughout the region, and the association was stronger when schizophrenia and BD-I were analyzed together. But BD-II showed a different pattern with few association signals.

The detailed genotypic association analysis results using logistic regression after controlling for the confounding effects of sex and age are summarized in S1 Table. Table 3 presents the results of the SNPs that showed an association with at least one diagnostic category with nominal significance. Statistical values are described only for the best-fit model. We observed nominally significant associations between schizophrenia and 10 SNPs under the additive model (rs4777969, rs4777973, rs11637898, rs4777980, rs8037133, rs2168351, rs3784731, rs1455777, rs897463, and rs7168443). These SNPs also showed associations under the dominant model, except rs3784731. rs2168351, rs3784731, and rs1455777 were also associated with schizophrenia under the recessive model. BD-I showed nominally significant associations with rs3759915, rs4777969, rs11637898, rs4777980, rs8037133, and rs2168351 under the dominant and additive models, and with rs8025225 under the recessive model. Nominally significant associations were observed between BD-II and rs3784737 (under the dominant model) and rs10775256 (under the recessive model). However, none of these associations remained significant after adjusting for multiple testing with Bonferroni’s correction.

**Table 3:** SNPs associated with either schizophrenia or bipolar disorder with nominally significant *p-*values (<0.05).

	Comparison with control group (N = 502)															
	Schizophrenia (N = 582)				BD-I (N = 180)				BD-II (N = 159)				Schizophrenia + BD-I (N = 762)			
SNP	P ^a^	OR	CI	Best-fit model ^b^	P ^a^	OR	CI	Best-fit model ^b^	P ^a^	OR	CI	Best-fit model ^b^	P ^a^	OR	CI	Best-fit model ^b^
rs3759915	0.245	0.843	0.632–1.124	Recessive	0.024*	1.609	1.065–2.430	Dominant	0.317	0.790	0.498–1.253	Recessive	0.102	1.236	0.959–1.594	Dominant
rs4777969	0.027*	0.812	0.675–0.977	Additive	0.032*	0.682	0.480–0.968	Dominant	0.213	0.785	0.537–1.149	Dominant	0.014*	0.806	0.678–0.957	Additive
rs8025225	0.412	0.870	0.623–1.215	Recessive	0.024*	0.519	0.294–0.916	Recessive	0.621	1.102	0.749–1.623	Dominant	0.131	0.782	0.569–1.076	Recessive
rs4777973	0.006*	1.420	1.107–1.820	Dominant	0.135	1.311	0.919–1.871	Dominant	0.439	1.162	0.794–1.701	Dominant	0.004*	1.405	1.113–1.774	Dominant
rs11637898	0.003*	1.443	1.130–1.843	Dominant	0.005*	1.641	1.162–2.319	Dominant	0.484	0.770	0.370–1.601	Recessive	0.0006**	1.492	1.186–1.878	Dominant
rs4777980	0.015*	1.261	1.047–1.520	Additive	0.002*	1.729	1.215–2.459	Dominant	0.134	0.550	0.251–1.203	Recessive	0.002*	1.440	1.143–1.814	Dominant
rs3784737	0.220	1.165	0.913–1.485	Dominant	0.343	1.182	0.837–1.670	Dominant	0.041*	1.485	1.017–2.170	Dominant	0.249	1.144	0.910–1.437	Dominant
rs8037133	0.026*	1.232	1.025–1.481	Additive	0.010*	1.591	1.117–2.264	Dominant	0.394	0.744	0.378–1.467	Recessive	0.006*	1.379	1.095–1.739	Dominant
rs2168351	0.011*	1.260	1.054–1.507	Additive	0.030*	1.483	1.038–2.120	Dominant	0.959	0.985	0.553–1.755	Recessive	0.007*	1.263	1.065–1.497	Additive
rs3784731	0.021*	1.656	1.080–2.540	Recessive	0.238	1.231	0.871–1.739	Dominant	0.583	0.900	0.619–1.310	Dominant	0.024*	1.227	1.027–1.466	Additive
rs1455777	0.014*	1.266	1.048–1.529	Additive	0.119	1.318	0.932–1.865	Dominant	0.731	0.936	0.643–1.363	Dominant	0.011*	1.261	1.055–1.508	Additive
rs10775256	0.178	0.775	0.535–1.123	Recessive	0.213	0.708	0.410–1.220	Recessive	0.039*	0.514	0.274–0.966	Recessive	0.125	0.763	0.539–1.078	Recessive
rs897463	0.011*	0.653	0.471–0.907	Additive	0.259	0.766	0.482–1.217	Recessive	0.490	0.464	0.052–4.102	Recessive	0.016*	0.688	0.507–0.932	Additive
rs7168443	0.029*	0.702	0.511–0.965	Additive	0.431	0.837	0.538–1.303	Recessive	0.316	0.780	0.480–1.267	Additive	0.048*	0.742	0.553–0.997	Additive

BD-I, bipolar I disorder; BD-II, bipolar II disorder; OR, odds ratio; CI, confidence interval

* *p* < 0.05

** corrected *p* < 0.05

^a^ Nominal *p*-value by logistic regression with age and sex covariates.

^**b**^ The inheritance model with the least Akaike Information Criterion was accepted as the best fitting model.

As the schizophrenia and BD-I groups showed similar association trends in the *ST8SIA2* region (Fig 1), and a previous linkage study defined these two disorders as a common locus phenotype for this region [9], we combined the two groups and applied the same association analysis. Ten SNPs revealed nominally significant associations with lower *p*-values compared to those of separate analyses for schizophrenia or BD-I. The association with rs11637898 reached the corrected level of significance under the dominant (*p* = 0.0006) and additive models (*p* = 0.0011). The minor allele of most of the SNPs showing significant associations was a risk allele for the corresponding illness, except rs4777969, rs8025225, rs897463, and rs7168443 for which the minor allele was a protective allele.

## Discussion

*ST8SIA2* is located on chromosome 15q25-26 and has been considered a positional and functional candidate gene for schizophrenia and BD, and significant associations have been reported in several different populations [12–15]. Genetic association or linkage with *ST8SIA2* has also been observed in patients with major depressive disorder [32, 33] and autism spectrum disorder [34]. These findings suggest that this gene has a role in the occurrence of major psychiatric disorders, which have varying degrees of neurodevelopmental defects.

**Fig 2:**
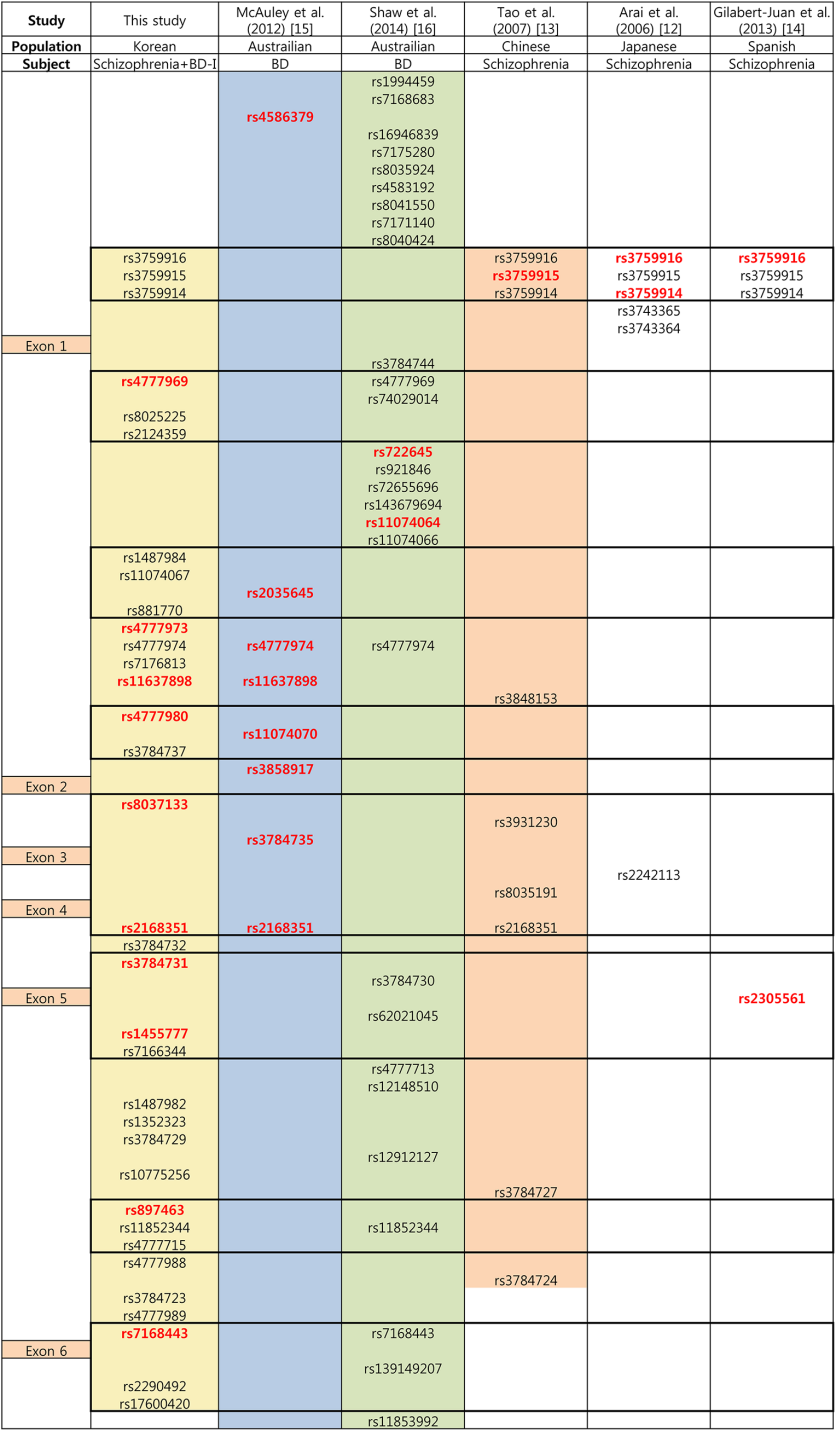
Relative positions of the *ST8SIA2* single nucleotide polymorphisms (SNPs) analyzed in the current study and previous studies reporting positive associations with schizophrenia or bipolar disorder. BD-I, bipolar I disorder; BD, bipolar disorder. Relative positions of the exons are displayed in the left column. Colored area is the covered area of the gene in the corresponding study. Box with bold outline indicates high linkage disequilibrium block (D’> 0.9) generated by Haploview v4.0 (http://www.broad.mit.edu/mpg/haploview) using the control group data of the current study (*n* = 502). SNPs with red letter indicate a significant association with nominal *p*-values < 0.05.

In the present study, we found suggestive associations between *ST8SIA2* and schizophrenia and BD-I in the Korean population. Through fine mapping of the gene with tag SNPs and additional candidate SNPs, we identified 14 associated variants with at least a nominal level of significance in a diagnostic group, however none of them survived after multiple testing correction; these association trends were strongest in the combined schizophrenia and BD-I group. The strongest association was observed between rs11637898 and schizophrenia and BD-I under the dominant model, and the association with combined schizophrenia and the BD-I group remained significant after correcting for multiple testing. These results are consistent with a previous study of the Australian population revealing an association trend between several SNPs in *ST8SIA2* and BD [15]. Two of these SNPs (rs11637898 and rs2168351) overlapped with SNPs showing association signals in the present study. Consistency between the two studies was also observed for two more pairs of SNPs with high linkage disequilibrium, i.e., rs4777980–rs11074070 and rs8037133–rs3784735. Fig 2 presents the relative positions of the *ST8SIA2* SNPs that have been studied in schizophrenia and/or BD.

Three SNPs selected from a putative promoter region (rs3759916, rs3759915, and rs3759914) showed an association with schizophrenia in the Japanese, Chinese, and Spanish populations [12–14]. In contrast, rs3759915 showed a nominally significant association with only the BD-I group in the current study.

A remarkable finding of the present study was that schizophrenia and only BD-I shared the same association pattern with *ST8SIA2* SNPs. This pattern was not observed in BD-II. As previous *ST8SIA2* genetic studies did not analyze BD-II separately, this result needs to be replicated in a future study. According to the DSM-IV or DSM-5, BD-I and BD-II are distinguished by the presence of manic or hypomanic episode. The criteria for mania and hypomania have the same symptom profile and differ only in the duration and severity of the episode. Thus, BD-II may be regarded as a milder form of BD-I in a spectrum of illnesses having different thresholds on a continuum of the same underlying multi-factorial vulnerability [35]. However, recent investigations suggest that BD-II could be a discrete diagnostic category from BD-I in genetic, biological, clinical, and pharmacological aspects [36–39]. Results of the present study seem to support differences in genetic make-up between patients with BD-I and BD-II.

In contrast, BD-I and schizophrenia showed the same *ST8SIA* association pattern. When we combined these two groups for the analysis, the significance of association became stronger. This finding is consistent with previous GWAS results suggesting common vulnerability genes in patients with schizophrenia and BD crossing the diagnostic boundary[40, 41]. Considering gene function, *ST8SIA2* could be related to the putative pathological mechanism of both schizophrenia and BD. Animal studies have shown that the amount of polySia synthesized by polysialyltransferase encoded by *ST8SIA2* is crucial during brain development [42, 43]. *ST8SIA2* knockout mice showed misguided infrapyramidal mossy fibers and formed ectopic synapses in the hippocampus [44]. These mice exhibited increased aggressive behavior and hyperactivity with impaired social behavior that could be seen in human patients with schizophrenia and BD [45]. In the context of gene-gene and gene-environment interactions, schizophrenia and BD-I may have some common vulnerability genes, and the susceptibility of each disorder could be established after the complex interplay of these genes with diagnosis-specific genes and environmental factors.

Another possible explanation for the common susceptibility between schizophrenia and BD-I is the existence of symptoms-specific genes rather than diagnosis-specific genes. Many attempts have been made to disassemble the diagnoses of schizophrenia and BD and reassess their symptoms in multiple phenomenological dimensions [46]. In the future, genetic studies targeting vulnerability genes for common clinical symptoms crossing the Kraepelinian dichotomy, such as psychotic features, mood dysregulation, and cognitive deficits, are needed.

The limitations of the current study are as follows. First, our sample size was relatively small, particularly for BD-II, which did not show the same significant association as BD-I. However, this negative result does not seem to be a false negative. The significant associations found in BD-I disappeared or became weaker when we analyzed the combined BD-I and BD-II group (S1 Table). Second, because we evaluated only diagnostic categories as phenotypes, we could not reveal possible associations of the gene with common clinical symptoms of schizophrenia and BD-I.

Considering both functional aspects and recent genetic study results including those of the present study, *ST8SIA2* may be a susceptibility gene for both schizophrenia and BD beyond the boundary of diagnosis. Schizophrenia and BD could be a clinical syndrome composed of various biological subgroups with heterogeneous genetic backgrounds and could share some genetic risks that affect early neurodevelopment. Reconstructing the phenotypes toward broader clinical categories crossing the current diagnoses, and toward symptom-based dimensions could guide us to novel findings and an understanding of the background behind major mental disorders.

## Supporting Information

S1 Table Detailed results of association analysis of *ST8SIA* tag single nucleotide polymorphisms with schizophrenia and bipolar disorders. (XLSX)
